# Evaluation of the effect of donor weight on adipose stromal/stem cell characteristics by using weight-discordant monozygotic twin pairs

**DOI:** 10.1186/s13287-021-02587-0

**Published:** 2021-09-26

**Authors:** Miia Juntunen, Sini Heinonen, Heini Huhtala, Aila Rissanen, Jaakko Kaprio, Kirsi Kuismanen, Kirsi H. Pietiläinen, Susanna Miettinen, Mimmi Patrikoski

**Affiliations:** 1 Adult Stem Cell Group, Faculty of Medicine and Health Technology, Tampere University, Arvo Ylpön katu 34, 33014 Tampere, Finland; 2 Research, Development and Innovation Centre, Tampere University Hospital, Tampere, Finland; 3 Obesity Research Unit, Research Program for Clinical and Molecular Metabolism, Faculty of Medicine, University of Helsinki, Helsinki, Finland; 4 Obesity Center, Abdominal Center, Endocrinology, University of Helsinki and Helsinki University Central Hospital, Helsinki, Finland; 5 Faculty of Social Sciences, University of Tampere, Tampere, Finland; 6 Institute for Molecular Medicine FIMM, HiLIFE, University of Helsinki, Helsinki, Finland; 7 Department of Obstetrics and Gynecology, Tampere University Hospital, Tampere, Finland

**Keywords:** Adipose stem cells, Cell surface markers, Differentiation, Immunogenicity, Immunosuppression, Proliferation

## Abstract

**Background:**

Adipose stromal/stem cells (ASCs) are promising candidates for future clinical applications. ASCs have regenerative capacity, low immunogenicity, and immunomodulatory ability. The success of future cell-based therapies depends on the appropriate selection of donors. Several factors, including age, sex, and body mass index (BMI), may influence ASC characteristics. Our aim was to investigate the effect of acquired weight on ASC characteristics under the same genetic background using ASCs derived from monozygotic (MZ) twin pairs.

**Methods:**

ASCs were isolated from subcutaneous adipose tissue from five weight-discordant (WD, within-pair difference in BMI > 3 kg/m^2^) MZ twin pairs, with measured BMI and metabolic status. The ASC immunophenotype, proliferation and osteogenic and adipogenic differentiation capacity were studied. ASC immunogenicity, immunosuppression capacity and the expression of inflammation markers were investigated. ASC angiogenic potential was assessed in cocultures with endothelial cells.

**Results:**

ASCs showed low immunogenicity, proliferation, and osteogenic differentiation capacity independent of weight among all donors. ASCs showed a mesenchymal stem cell-like immunophenotype; however, the expression of CD146 was significantly higher in leaner WD twins than in heavier cotwins. ASCs from heavier twins from WD pairs showed significantly greater adipogenic differentiation capacity and higher expression of *TNF* and lower angiogenic potential compared with their leaner cotwins. ASCs showed immunosuppressive capacity in direct cocultures; however, heavier WD twins showed stronger immunosuppressive capacity than leaner cotwins.

**Conclusions:**

Our genetically matched data suggest that a higher weight of the donor may have some effect on ASC characteristics, especially on angiogenic and adipogenic potential, which should be considered when ASCs are used clinically.

**Supplementary Information:**

The online version contains supplementary material available at 10.1186/s13287-021-02587-0.

## Introduction

Adipose stromal/stem cells (ASCs) are multipotent mesenchymal stromal/stem cells (MSCs) that represent a promising tool for tissue regeneration applications as well as for cell-based treatment of inflammatory and autoimmune conditions [1]. The International Society for Cellular Therapy (ISCT) has stated minimal definitions for MSCs [2]. MSCs are heterogeneous, plastic adherent cells, which express CD90, CD73, and CD105 and lack the expression of CD45, CD34, CD14 or CD11b, CD79α or CD19, and HLA-DR and differentiate into adipogenic, osteogenic, and chondrogenic lineages in vitro [2]. The success of future cell-based therapies may depend on the appropriate selection of donors. Several factors of the donor, such as age [3–5] and sex [6], have been suggested to affect ASC characteristics. For instance, the osteogenic differentiation [4–6] and proliferation [7] capacity of ASCs have been shown to differ according to donor properties. Moreover, culturing conditions may influence ASC characteristics [8], and the passaging of cells may alter the ASC immunophenotype as the cell population becomes more homogenous [9, 10].

The World Health Organization estimated in 2016 that more than 1.9 billion adults are overweight (39%), of which 650 million are obese (13%) [11]. Excess weight gain leads to overweight and obesity, which causes inflammation in adipose tissue (AT). Obesity changes the metabolic and endocrine functions of AT, leading to various metabolic diseases, such as insulin resistance and type II diabetes [12], cardiovascular diseases, musculoskeletal disorders and some cancers [13]. The influence of obesity has become an interesting question for cell therapy, since in recent studies, it has been shown that obesity and chronic inflammation may affect ASC characteristics and functionality [14, 15].

ASCs isolated from donors with obesity (obASCs) may have altered immunophenotypes [16, 17]. Furthermore, the proliferation capacity of obASCs may be either increased [16, 18] or decreased compared with that of ASCs isolated from lean donors (lnASCs) depending on the study [17, 19–22], and in obASCs, senescence-associated gene expressions may be upregulated [22]. Most of the studies have shown a decreased differentiation capacity of obASCs. The osteogenic capacity of obASCs has been shown to decrease compared with lnASCs both in vivo [23] and in vitro [17, 19, 23], and adipogenic differentiation capacity has been shown to decrease in vitro [16, 17, 21, 24, 25]. However, regarding both osteogenic [26, 27] and adipogenic [18, 19, 26] differentiations, opposing results exist in which the differentiation capacity of obASCs has either increased [18, 26] or has not been affected [19, 27] compared to lean donors. Moreover, obASCs may have a reduced capacity for immunosuppression compared with lnASCs [28, 29]. These studies have brought into question whether obASCs should be used in tissue engineering applications [23] or as a cell-based therapy for inflammatory diseases [29].

Since the previous results are partly contradictory, the effect of increased weight on ASCs should be investigated in more detail taking the genetics of the donor into account. Our present study aims to clarify whether controlling for genetic background affects the association of weight with ASC characteristics. We used rare donor material of weight-discordant (WD) monozygotic (MZ) adult twin pairs [30]. The aim of the current study was to investigate the effect of intrapair differences in weight on basic ASC characteristics such as the proliferation and differentiation capacity, immunological characteristics, and ASC angiogenic potential while controlling for genetic variation between the lean and heavy groups.

## Materials and methods

### Weight-discordant MZ twin donors

This study included five WD (within-pair difference in body mass index (BMI) > 3 kg/m^2^) MZ twin pairs (age = 33.5–34.1 years at time of the study, males *n* = 6, females *n* = 4), from two population-based large twin cohorts, namely FinnTwin16 and FinnTwin12 (*n* = 5417 pairs). The twin pairs have previously been part of a larger metabolic study [30]. MZ twins were categorized into two groups: leaner cotwins (*n* = 5, mean BMI 26.4 ± 7.7 kg/m^2^) and heavier cotwins (*n* = 5, mean BMI 31.6 ± 7.9 kg/m^2^; Table 1). The term cotwin is used when referring to a birth partner of a MZ twin. Written informed consent was obtained from all participants. The study was conducted in accordance with the Ethics Committee of Hospital District of Helsinki and Uusimaa, Helsinki, Finland (270/13/03/01/2008).

**Table 1:** Clinical characteristics of the MZ twins

Variable	Weight-discordant pairs (ΔBMI > 3 kg/m^2^)						
	Leaner cotwin			Heavier cotwin			*p* Value
	Median	Min	Max	Median	Min	Max	
BMI	23.3	21.5	39.9	26.8	25.8	44.2	**0.043**
Cholesterol (mmol/l)	3.9	3.0	4.7	5.0	3.0	6.9	0.276
Triglycerides (mmol/l)	0.6	0.5	0.8	1.4	0.5	2.7	0.225
HDL (mmol/l)	1.7	1.3	2.1	1.3	0.5	2.2	0.138
LDL (mmol/l)	2.0	1.6	3.0	3.4	1.6	4.5	0.080
hs-CRP (mg/l)	0.4	0.2	3.9	0.8	0.2	7.0	0.225
HOMA-IR	0.8	0.3	1.0	1.8	0.6	2.7	0.138
Plasma leptin (ng/ml)	10.0	0.7	77.6	22.1	5.2	55.7	0.500
Plasma adiponectin (ng/ml)	5050	3700	7710	4030	1310	4930	**0.043**

Wilcoxon’s signed-rank test was used to compare the values of the leaner vs heavier cotwin. A *p* value less than 0.05 was considered significant (shown in bold). HDL = high-density lipoprotein, LDL = low-density lipoprotein, hs-CRP = high-sensitivity C reactive protein, HOMA-IR = insulin resistance index

### Measurements of biochemical variables

Weight and height were measured after a 12-h overnight fast while barefoot and in light clothing to calculate BMI. Fasting blood samples were taken from all donors to measure clinical parameters as follows. The concentrations of serum high-sensitivity C reactive protein (hs-CRP) were measured by a particle-enhanced immunoturbidimetric assay (Cobas CRP [latex] HS, Roche Diagnostics, Mannheim, Germany) on a modular automatic analyzer (Hitachi, Ltd., Tokyo, Japan). Plasma leptin and adiponectin were measured by enzyme-linked immunosorbent assays (ELISA; DuoSet ELISA, R&D Systems Europe, Abingdon, UK), and plasma total and high-density lipoprotein (HDL) cholesterol and triglyceride levels were measured by enzymatic methods (Roche Diagnostics Hitachi, Hitachi Ltd, Tokyo, Japan). Low-density lipoprotein (LDL) cholesterol was calculated by the Friedewald formula. A 75-g oral glucose tolerance test was performed after participants had fasted for 12-h overnight to calculate the insulin resistance index (HOMA-IR). Zygosity was confirmed by genotyping 10 informative genetic markers [31].

### ASC isolation and culture

Subcutaneous AT was biopsied from both twins in MZ twin pairs, and ASCs were isolated from AT and maintained as previously described [32]. ASCs were cultured in DMEM/F12 (1:1; Thermo Fisher Scientific Inc., Carlsbad, CA) supplemented with 5% human serum (HS; Paa Laboratories/BioWest) or 5% pooled human platelet lysate (HPL; Stemulate™; Cook General BioTechnology, Indianapolis, IN), 1% antibiotics (100 U/ml penicillin and 0.1 mg/ml streptomycin; Thermo Fisher Scientific Inc.), and 1% L-glutamine (GlutaMAX-100; Thermo Fisher Scientific Inc.; see Additional file 1: Table 1). Differentiation of ASCs and angiogenesis assays was assessed in HS medium and other analyses in HPL medium. The medium was changed twice a week, and the cells were divided upon reaching confluency. Cells were detached using TrypLE Select (Thermo Fisher Scientific Inc.).

### Immunophenotype

The phenotype of ASCs was assessed with flow cytometry (BD FACSAria™ Fusion Cell Sorter; BD Biosciences) at passage 5. Monoclonal antibodies against CD markers (see Additional file 1: Table 2) CD19-phycoerythrincyanine (PE-Cy7), CD14- PE-CF594, CD34-PE-CF594, CD45RO- allophycocyanin (APC), CD54-brilliant violet 711 (BV711), CD73-PE-Cy7, CD90-APC, CD146-PE, HLA-DR-BV421 (BD Biosciences), CD105-fluorescein isothiocyanate (FITC), and HLA-ABC-PE (ImmunoTools GmbH, Friesoythe, Germany) were used. Multicolor staining was used to assess the immunophenotype of ASCs. Ten thousand cells were analyzed, and unstained cells were used to adjust the cytometer. Compensation for all the antibodies was performed using Compensation Plus beads (BD Biosciences).

### Proliferation

The proliferation of ASCs was assessed with the Cell Counting Kit-8 assay (CCK-8; Dojindo Laboratories, Japan) as previously described [33] at passage 4 or 5. Briefly, cells were seeded in 48-well plates (Nunc, Thermo Fisher Scientific Inc.) at a density of 260 cells/cm^2^, and proliferation was measured at 4, 7, and 11 days in HPL medium. Culture medium was discarded, wells were rinsed with Dulbecco’s phosphate-buffered saline (DPBS; Lonza, BioWhittaker, Verviers, Belgium), and proliferation was measured using a microplate reader (Victor 1429 Multilabel Counter, Wallac, Turku, Finland) at 450 nm after incubating cells for 3 h in DPBS and CCK-8 reagent 10:1 at 37 °C in a CO_2_ incubator (Heracell).

### Osteogenic differentiation, mineralization, and alkaline phosphatase activity

For osteogenic differentiation capacity studies, ASCs were seeded in 24-well plates (Corning® CELLBIND® Corning Inc., Corning, NY, USA) at a density of 260 cells/cm^2^ at passage 3 or 4. ASCs were cultured in osteogenic medium (OM; see Additional file 1: Table 1) for 21 days for Alizarin red (AR) staining, and quantification (QAR) was performed as previously described [34]. To study the early osteogenic differentiation of ASCs, ASCs were cultured in OM for 14 days. Alkaline phosphatase (ALP) activity measurements were performed, and the results were normalized to the DNA amount from CyQUANT analysis from the same cell lysates as previously described [34]. DNA amount measurement was conducted with a CyQUANT™ cell proliferation assay kit (Molecular Probes, Invitrogen™, Paisley, UK) according to the manufacturer’s protocol.

### Adipogenic differentiation and quantification of lipid formation

For adipogenic differentiation capacity studies, ASCs were seeded in 24-well plates (Corning® CELLBIND®) at a density of 260 cells/cm^2^ at passage 3 or 4. ASCs were cultured in adipogenic medium (AM; see Additional file 1: Table 1) for 21 days, and Oil Red O staining (ORO) was performed as previously described [35]. Cells were photographed with a fluorescence microscope (Nikon Olympus), and lipid formation was analyzed using quantified ORO (QORO) and DAPI images as previously described [35] using CellProfiler software (version 2.1.1, 64-bit Windows). Lipid formation was evaluated by applying a 10-μm-diameter threshold to identify large lipid droplet (LD) clusters.

### qRT-PCR for osteogenic, adipogenic, and inflammatory markers

The relative expression of the osteogenic genes *Runt-related transcription factor 2* (*RUNX2*) and *ALP* and the adipogenic genes *peroxisome proliferator-activated receptor gamma* (*PPARγ*) and *adipocyte protein 2* (*AP2*) (see Additional file 1: Table 3 for primer sequences) was assessed as previously described [36]. Gene expression was performed with Power SYBR Green PCR Master Mix (Applied Biosystems, Warrington, Cheshire, UK) on an ABI Prism 7300 real-time PCR system (Thermo Fisher Scientific). The housekeeping gene *ribosomal phosphoprotein P0* (*RPLP0*) was used as an internal control, and the relative expression of the studied genes was calculated using a mathematical model as previously described [37]. The expression levels of the inflammatory markers *interleukin 6* (*IL-6*) and *tumor necrosis factor* (*TNF*) were analyzed with TaqMan Gene Expression Assays (Applied Biosystems) using an ABI Prism 7300 real-time PCR system (see Additional file 1: Table 4). The results were calculated using the delta Ct method using *glyceraldehyde-3-phosphate dehydrogenase (GAPDH)* as an endogenous control.

### Chondrogenic differentiation

Chondrogenic differentiation was conducted at passage 5 or 6 as previously described (10,38) in micromass cultures. Briefly, 8 × 10^4^ ASCs were seeded into 24-well plate in a 10-µl volume and cells adhered for 3 h at 37 °C in a CO_2_ incubator. Thereafter, ASCs were cultured in chondrogenic medium (CM, see Additional file 1: Table 1) for 14 days. After the chondrogenic differentiation, Alcian blue staining was performed as previously described [38]. Briefly, cell pellets were fixed with 4% PFA and stored in 70% ethanol. After fixing, pellets were dehydrated, embedded into paraffin, and sectioned at 4 µm thickness. The sections were rehydrated and stained with Alcian blue (pH 1.0) and counterstained with Nuclear Fast Red solution (Sigma-Aldrich, Merck KGaA, Darmstadt, Germany). Samples were photographed using Zeiss Axio Vert.A1 microscope with Zeiss AxioCam MRC5.

### Angiogenesis potential assay

For the angiogenesis potential assay, human umbilical vein endothelial cells (HUVECs) were isolated from the umbilical cord received from a planned cesarean section at the Department of Obstetrics and Gynecology, Tampere University Hospital, with donor consent. The study was conducted in accordance with the Ethics Committee of the Pirkanmaa Hospital District, Tampere (R13019). HUVEC isolation was performed as previously described [39]. HUVECs were cultured in endothelial medium (EM; Lonza) supplemented with 2% HS (BioWest, see Additional file 1: Table 1). For the angiogenesis potential assay, ASCs were plated in 48-well plates (Nunc) at a density of 23,000 cells/cm^2^ at passage 4 or 6, and HUVECs were plated at a density of 4600 cells/cm^2^ at passage 4 in EM after 3 h of attachment of ASCs. Cells were cocultured for 5 days and stained with 1:200 anti-collagen type IV (Domain, clone IV-4H12) mouse monoclonal antibody (Millipore) and goat anti-mouse IgG1 cross-adsorbed secondary antibody Alexa 488 (Thermo Fisher Scientific). Stained cells were photographed using Cell-IQ (CM Technologies Oy, Tampere, Finland) with a 10 × objective and 3 × 3 grid to detect vascular structures. Grids were stitched together with a Cell-IQ Analyzer (CM Technologies Oy), and quantitative analysis of angiogenesis was performed using AngioTool64 Version 0.6a (02.18.14) [40].

### Immunogenicity

For immunogenicity and immunosuppression capacity studies, peripheral blood mononuclear cells (PBMCs) were isolated from buffy coat samples and frozen as previously described [8]. The buffy coat samples were obtained from the Finnish Red Cross Blood Service, and the study was conducted in accordance with the Declaration of Helsinki 1975, revised in Hong Kong 1989. The immunogenicity of ASCs was studied using a one-way mixed lymphocyte reaction (MLR) as previously described [8]. Briefly, irradiated ASCs at a density of 62,500 cells/cm^2^ at passage 4 or 5 were cocultured with PBMCs at a density of 625,000 cells/cm^2^ in 96-well plates (Nunc) for 5 days in HPL medium. For nonproliferative control samples, PBMCs were irradiated to prevent cell proliferation, and for proliferative control samples, PBMCs were cultured with 10 µg/ml phytohemagglutinin-M (PHA; Sigma-Aldrich) to enhance proliferation. The proliferation of PBMCs was measured with BrdU ELISA (Roche Applied Science, Penzberg, Germany).

### Immunosuppression capacity

The immunosuppressive capacity of ASCs was studied using two-way MLR in both direct and indirect cocultures as previously described [8]. Briefly, ASCs were pretreated with 10 ng/ml interferon-γ (Sigma-Aldrich) for 72 h to enhance the immunosuppressive capacity of ASCs. ASCs were plated in 24-well plates (Nunc) at a density of 63,000 c/cm^2^ or in ThinCerts™-TC inserts (Greiner bio-one, Switzerland) at a density of 360,000 cells/cm^2^ at passage 4, 5 or 6. A mixture of two PBMC donor cell lines was plated in wells at a density of 630,000 cells/cm^2^. ASC and PBMC mixtures were also cultured alone as control samples. After 5 days of coculture in HPL medium, the proliferation of PBMCs was measured with the BrdU ELISA.

### BrdU ELISA for lymphocyte proliferation

The proliferation of PBMCs was assessed with the BrdU ELISA 5 days after cells were plated in wells for immunogenicity and immunosuppression capacity studies according to the manufacturer’s instructions. Calculations were performed from raw absorbance data of BrdU ELISA as previously described [8]. Briefly, average absorbance values were calculated for each group. The average absorbance of ASCs alone was subtracted from the MLR average values for each well. Thereafter, MLR values were divided by the average autologous PBMC value, which was considered the baseline response in one-way MLR, or MLR values were divided by the average PBMC mixture value without ASCs, which was considered the baseline response in two-way MLR. According to the performed calculations, in one-way MLR, a value of 1 indicates the baseline response, and values > 1 indicate activation; in two-way MLR, a value of 1 represents a baseline response, and reaction values < 1 indicate suppression. The performed calculations were modified from McIntosh et al. [41].

### Statistical analyses

The differences in mineralization (QAR), relative expression of *ALP*, proinflammatory markers *IL-6* and *TNF*, adipogenic differentiation (large LD formation), angiogenic potential (vasculature area and length) and immunosuppression capacity in direct cocultures between leaner WD twins compared with heavier WD cotwins were assessed using multilevel mixed-effects regression models to account for the nested data structure (i.e., multiple measurements within twin and pairing of the cotwins). To statistically test whether leaner and heavier WD cotwins differ in clinical parameters and surface marker expression of ASCs, the Wilcoxon signed-rank test was used. A *p* value less than 0.05 was considered significant. Data on clinical parameters and surface marker expression are presented as the median and the min and max values, and other data are presented as the mean ± standard deviation (SD). Statistical analyses were performed using Stata statistical software (Release 13.0, Stata Corporation, College Station, TX, USA), and graphs were generated using GraphPad Prism 5.02 (GraphPad Software, Inc.).

## Results

### Within WD twin pairs, heavier cotwins have more impaired metabolic health

As per design, the BMI of heavier WD cotwins was significantly higher than that of leaner WD twins (*p* = 0.0431). In four out of five WD twin pairs, heavier cotwins showed elevated levels of LDL, hs-CRP, and leptin and impaired insulin sensitivity measured as HOMA-IR compared with leaner cotwins. None of the donors satisfied the criteria for type 2 diabetes (Table 1). In addition, in four out of five WD twin pairs, the heavier cotwins showed lower levels of HDL, and all heavier WD twins showed significantly lower plasma adiponectin (*p* = 0.0431) than leaner WD cotwins (Table 1).

### ASCs showed an MSC-like immunophenotype independent of weight difference, except for CD14 and CD146

ASCs showed a characteristic MSC-like surface marker expression profile and morphology. ASCs showed high median expression (> 99%) of the surface markers CD73, CD90, CD105, and HLA-ABC (Table 2). The median expression of CD14, CD19, CD45RO, and HLA-DR was very low (≤ 2%), the median expression of CD34 was low (> 2 to < 7%), and the median expression of CD54 was moderate (> 11 to < 21%; Table 2). However, surface marker expression was similar between leaner and heavier WD twins, except for the expression of the surface markers CD14 and CD146, whose expression was significantly higher in all leaner twins than in heavier cotwins (*p* = 0.0394 and *p* = 0.0431, respectively, Table 2). The results of surface marker expression of ASCs isolated from each MZ twin donor are shown in Additional file 1: Table 5 and morphology in Additional file 7: Figure 6.

**Table 2:** Surface marker expression of ASCs isolated from MZ twin pairs at passage 5

Surface marker	Weight-discordant pairs (ΔBMI > 3 kg/m^2^)						
	Leaner cotwin			Heavier cotwin			*p* Value
	Median	Min	Max	Median	Min	Max	
CD14	0.3	0.3	0.5	0.2	0.2	0.3	**0.039**
CD19	1.5	0.9	2.9	1.6	1.0	3.1	0.588
CD34	2.3	2.1	8.3	7.1	1.8	9.8	0.345
CD45RO	0.9	0.6	1.0	0.9	0.7	1.4	0.219
CD54	11.4	4.0	35.7	13.1	4.0	24.8	0.276
CD73	100	99.9	100	100	99.9	100	ND
CD90	99.7	96.6	100	99.7	99.7	100	0.090
CD105	99.3	99.1	99.7	99.2	95.1	99.7	0.276
CD146	45.0	5.2	71.3	14.5	5.0	33.8	**0.043**
HLA-DR	0.7	0.3	6.4	0.4	0.2	0.4	0.138
HLA-ABC	99.6	98.2	99.7	99.5	99.3	99.9	0.343

Wilcoxon’s signed-rank test was used to compare values of the leaner vs the heavier cotwin. A P value less than 0.05 was considered significant (shown in bold). ND = not detected

### Acquired weight did not affect the proliferation of ASCs

**Fig. 1:**
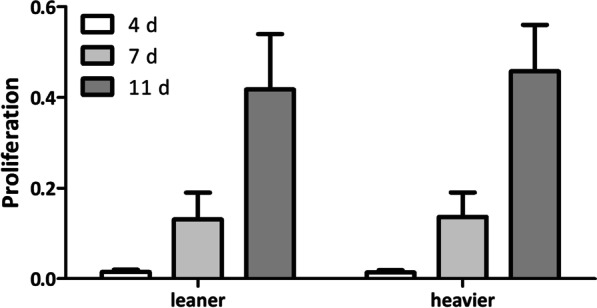
Proliferation of ASCs after 4, 7, and 11 days of culture in HPL medium. Leaner and heavier twin pairs show similar proliferation capacities. Data are presented as the average ± SD

The proliferation of ASCs was studied using a CCK-8 kit after 4, 7, and 11 days of culturing ASCs in HPL medium. Proliferation was similar between leaner and heavier WD cotwins (Fig. 1), but more variability was seen between pairs (see Additional file 2: Fig. 1).

### Osteogenic differentiation capacity of ASCs showed characteristic donor variation that was not dependent on acquired weight

**Fig. 2:**
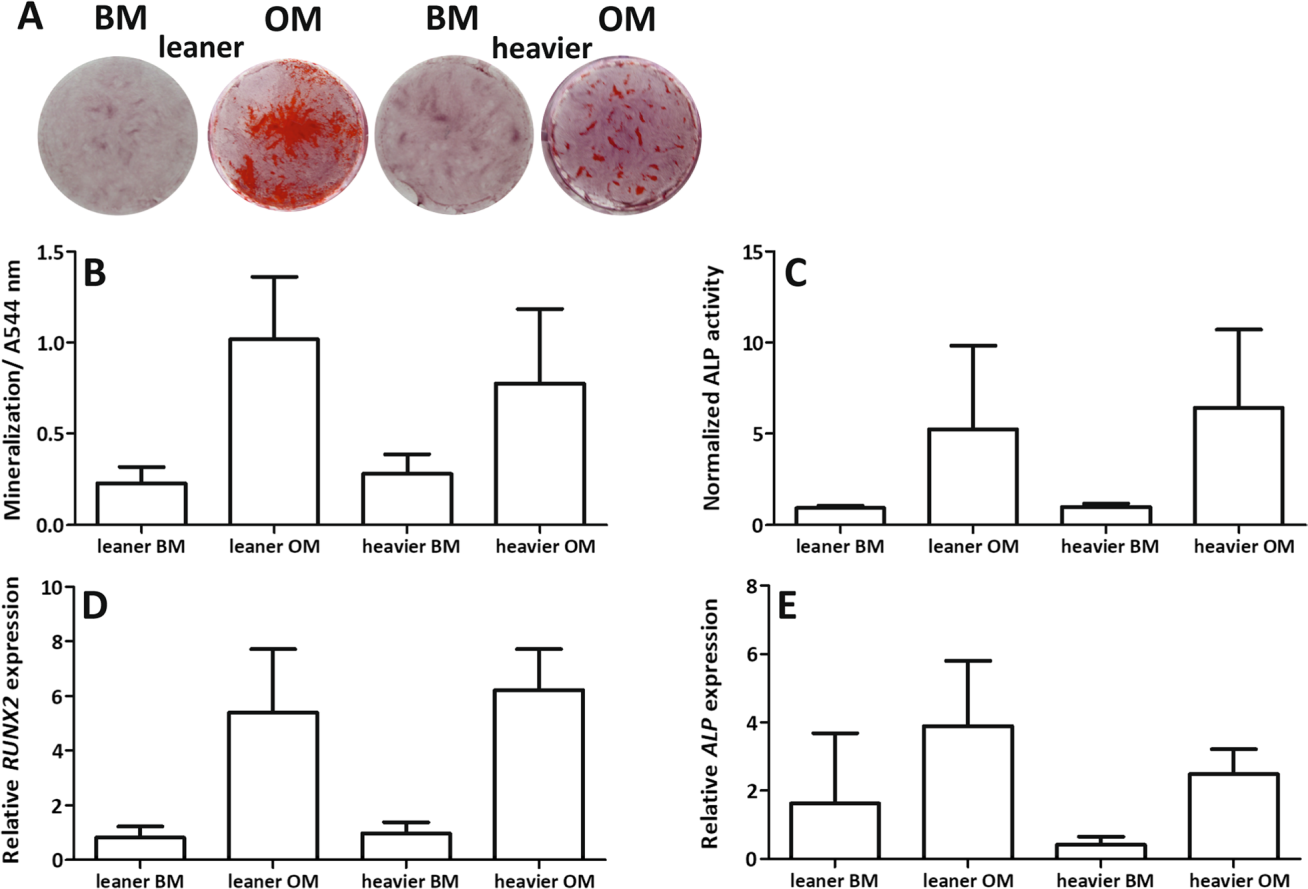
Osteogenic differentiation capacity of ASCs. **A** Representative image of Alizarin red staining, **B** quantified Alizarin red staining, **C** normalized alkaline phosphatase activity (ALP), **D** relative gene expression of *RUNX2* (d) and **E** *ALP*. Quantitative data presented as the average ± SD. BM = HS medium, OM = osteogenic medium

The osteogenic capacity of ASCs was studied after 14 days of osteogenic differentiation. QALP analysis showed similar ALP activity with both leaner and heavier WD twins (Fig. 2c). However, the relative expression of *ALP* was higher in three out of four leaner WD twins than in heavier WD cotwins (see Additional file 3: Fig. 2e). In addition, the relative expression of *RUNX2* showed similar expression in both leaner and heavier WD twins (Fig. 2d).

AR staining was performed after 21 days of osteogenic differentiation to study the late osteogenic differentiation capacity and mineralization of ASCs. AR staining showed the mineralization capacity of ASCs with all twin pairs under osteogenic differentiation conditions (Additional file 3: Fig. 2a). A representative image of AR staining is shown in Fig. 2a. The mineralization capacity was lower in three out of four heavier WD twins than in the leaner WD cotwins (Fig. 2b). The osteogenic differentiation capacity results of ASCs isolated from each donor can be found in Additional file 3: Fig. 2.

ASCs from both leaner and heavier WD twins was able to differentiate toward chondrogenic lineage, as shown by Alcian blue staining (see Additional file 8: Fig. 7).

### Heavier WD twin ASCs had significantly more large LD than ASCs isolated from leaner cotwins

**Fig. 3:**
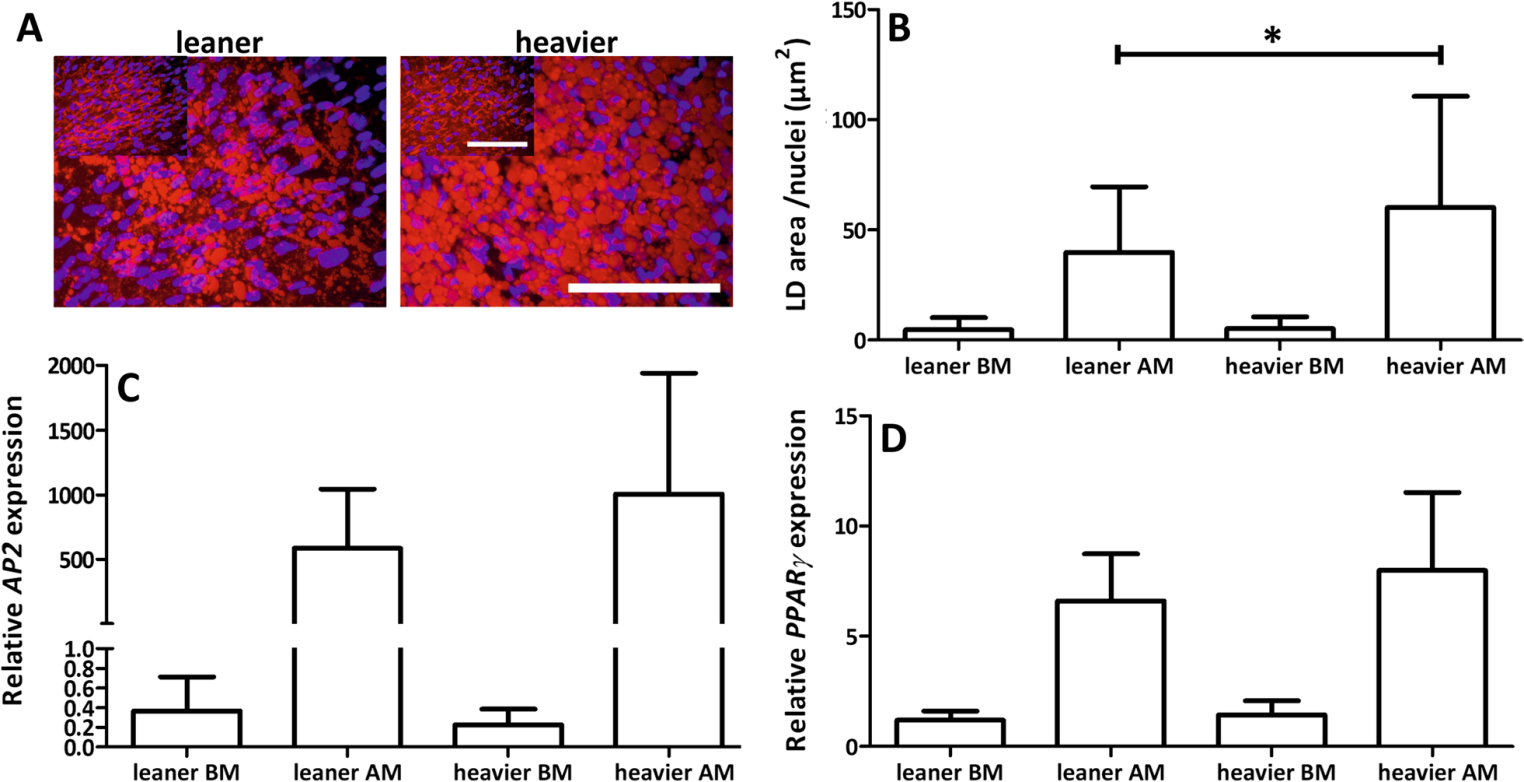
Adipogenic differentiation capacity. **A** Representative image of Oil Red O staining (ORO), scale bar 200 µm, inset = undifferentiated ASCs, larger picture = ASCs after 14 days of adipogenic differentiation, **B** quantified ORO staining (QORO) and lipid droplet (LD) area/nuclei (µm^2^), **C** relative gene expression of *AP2* and **D** *PPARγ*. Quantitative data presented as the average ± SD. BM = HS medium, AM = adipogenic medium. Statistical analyses were performed using multilevel mixed-effects regression model test for the nested data structure for QORO and Wilcoxon signed-rank test for relative gene expression of *AP2* and *PPARγ.* Statistical significance: * = *p* < 0.05. All quantitative data are presented as the mean ± SD

The adipogenic capacity of ASCs was studied after 14 days of adipogenic differentiation. ORO staining showed that ASCs isolated from heavier WD twins had higher differentiation capacity toward the adipogenic lineage than ASCs isolated from leaner cotwins (Fig. 3a, see Additional file 4: Fig. 3a). A representative image of ORO staining is shown in Fig. 3a. QORO showed that ASC isolated from heavier WD twins had a significantly higher number of large LD than ASC isolated from leaner WD cotwins (*p* = 0.011, Fig. 3b). However, the relative expression of *AP2* and *PPARγ* after 14 days in adipogenic conditions showed similar gene expression between leaner and heavier WD twins (Fig. 3c,d), except for one twin pair in which the heavier cotwin showed higher expression of *AP2* and *PPARγ* (Additional file 4: Figure S3c,d). The adipogenic differentiation capacity results of each individual ASC donor are presented separately in Additional file 4: Fig. 3.

### ASCs from leaner WD twins showed higher angiogenic potential

**Fig. 4:**
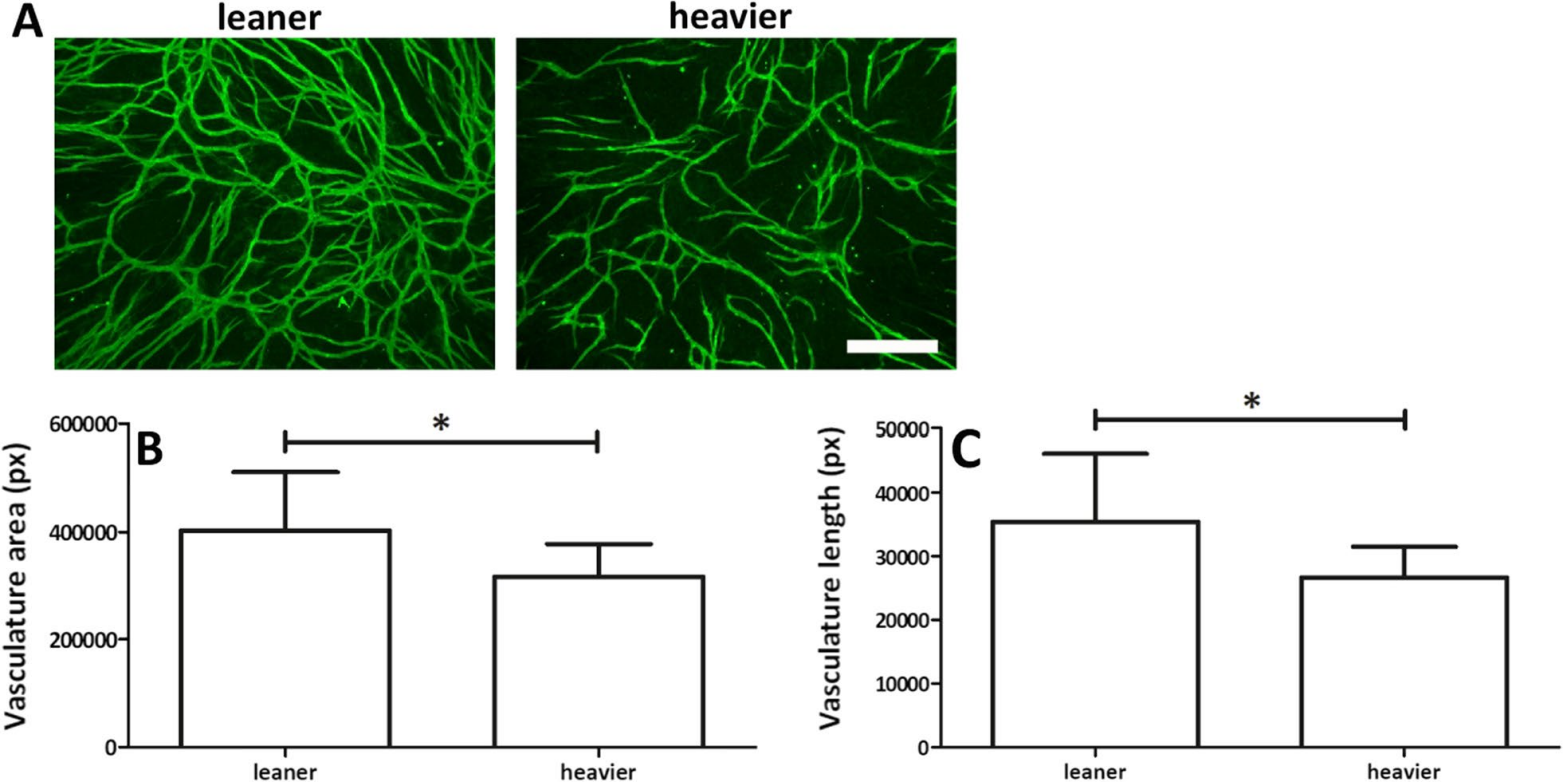
Angiogenic potential of ASCs. **A** Representative image of collagen type IV staining of ASC and HUVEC cocultures shows the formation of vasculature-like structures, scale bar 500 µm. **B** Vasculature area and **C** vasculature length. All statistical analyses were performed using multilevel mixed-effects regression model test for the nested data structure. Statistical significance: * = *p* < 0.05. Quantitative data presented as the average ± SD

Collagen type IV staining of ASC and HUVEC cocultures showed that HUVECs formed vasculature-like structures with both leaner and heavier WD twins (Fig. 4a). The image analysis confirmed that leaner WD twins showed a 21% larger area (*p* = 0.039, Fig. 4b) and 25% longer vasculature-like structures (*p* = 0.031, Fig. 4c) than heavier cotwins. The vasculature area and length of the vasculature-like structures formed from each donor can be found in Additional file 5: Fig. 4.

### ASCs showed low immunogenicity independent of intrapair weight differences; however, heavier WD donors showed superior immunosuppression capacity

**Fig. 5:**
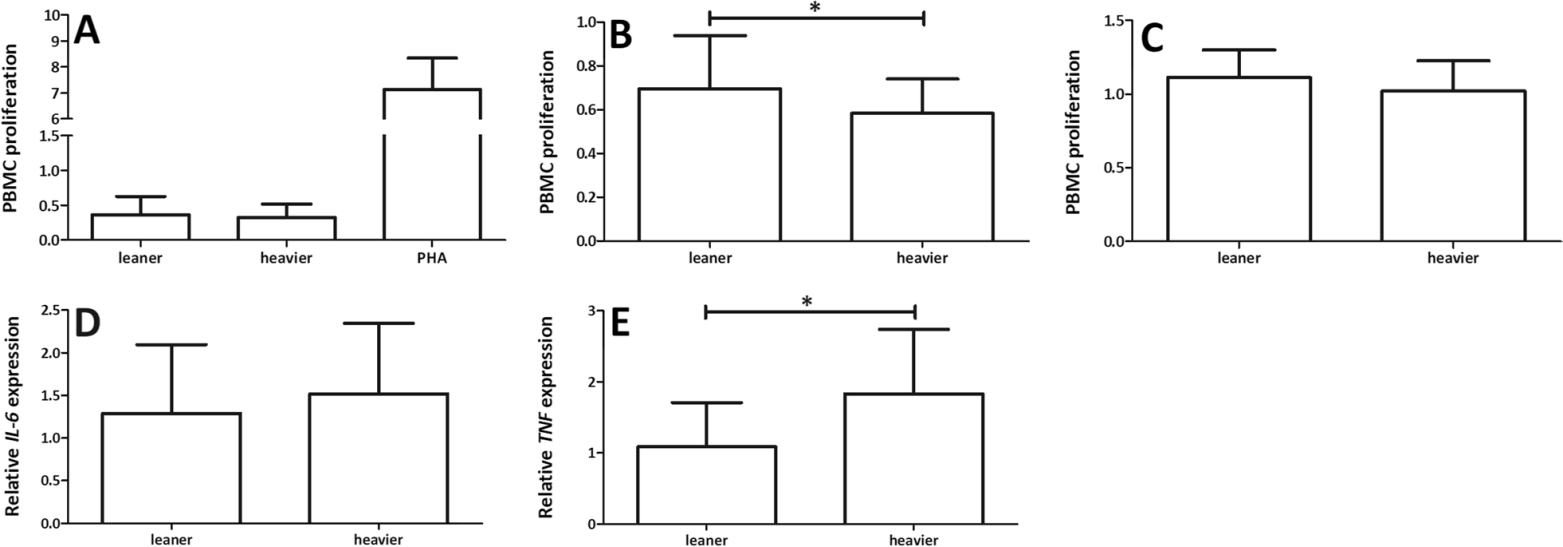
**A** Immunogenicity of ASCs derived from MZ twin donors using one-way MLR analysis. Peripheral blood mononuclear cell (PBMC) proliferation after 5 days of coculturing ASCs with PBMCs. Phytohemagglutinin-M (PHA). **B** Immunosuppressive capacity of ASCs derived from MZ twin donors in direct cocultures with PBMCs. PBMC proliferation was suppressed in direct cultures but not in **C** indirect cultures. **D** Relative gene expression of *IL-6* and **E** *TNF*. Statistical analyses were performed using multilevel mixed-effect regression model test for the nested data structure for immunosuppression in direct cocultures and relative gene expression of *IL-6* and *TNF*, and Wilcoxon signed-rank test for immunogenicity and immunosuppression in indirect cocultures. Significance: * = *p* < 0.05. All quantitative data are presented as the mean ± SD

The immunogenicity of ASCs was studied using one-way MLR, and the proliferation of PBMCs was measured after 5 days of coculturing ASCs with PBMCs. ASCs isolated from both WD leaner and heavier cotwins has low immunogenicity, which was assessed as low PBMC proliferation in cocultures with ASCs. PHA-stimulated PBMCs represents a positive control (Fig. 5a). The immunogenicity results of each donor can be found in Additional file 6: Fig. 5a.

The immunosuppressive capacity of ASCs was studied using two-way MLR in both direct and indirect cocultures. The results showed that ASCs isolated from both leaner and heavier WD twins suppressed PBMC proliferation in direct two-way MLR (Fig. 5b). The immunosuppressive capacity of ASCs significantly differed within WD twin pairs in direct cocultures. In four out of five WD twin pairs, the heavier cotwins showed improved suppression capacity compared with the leaner cotwins (*p* = 0.017, Fig. 5b). In indirect coculture, ASCs derived from both leaner and heavier WD twin donors did not suppress PBMC proliferation (Fig. 5c). Suppression of PBMC proliferation was more effective in direct culture than in indirect culture (Fig. 5b,c). Immunosuppression results of ASCs isolated from each donor can be found in Additional file 6: Fig. 5b, c.

### Within WD twin pairs, ASCs from heavier cotwins showed significantly elevated expression of the inflammatory marker *TNF*

The relative expression of the inflammatory markers *IL-6* and *TNF* was analyzed in ASCs after 14 days of culture in HS medium. The relative expression of *IL-6* was slightly elevated in heavier WD twins than in leaner cotwins (Fig. 5d). However, the relative expression of *TNF* was significantly elevated compared with that in leaner WD cotwins (*p* = 0.015, Fig. 5e). The expression of inflammatory markers from each donor can be found in Additional file 6: Fig. 5d, e.

## Discussion

To our knowledge, the current study is the first to investigate the effect of intrapair weight differences on ASC characteristics using rare MZ WD twin pairs with controls for genetic backgrounds. Our material consisted of 10 donors comprising five MZ WD twin pairs with intrapair weight differences ranging from 12 to 27 kg, which is exceptional for MZ pairs in general [42].

Several biochemical variables were measured from plasma samples of MZ twin pairs to characterize the current metabolic health of the donors. As expected, heavier WD cotwins had higher LDL, hs-CRP, insulin resistance, and leptin levels and lower HDL and adiponectin levels than leaner WD cotwins, as has been reported before for weight-discordant MZ pairs [30]. In general, all donors were clinically and even metabolically rather healthy independent of BMI status. As relatively young adults, even obese twins have not yet developed clinical complications and therefore represent an ideal sample to study the effects of adiposity alone on ASC characteristics.

Previous studies have demonstrated that obesity alters ASC characteristics such as proliferation capacity [15]. Most of the studies have shown reduced proliferation capacity of obASCs compared with lnASCs [17, 19–21], although opposing results of elevated obASC proliferation have also been reported [16, 18]. Our results showed that the acquired weight of the donor compared to his/her leaner genetically matched cotwin does not affect the proliferation capacity of ASCs. Similar findings have been reported earlier by Mojallal et al. [43], who showed that BMI and proliferation did not significantly correlate. In the current study, the genetic and other shared backgrounds seemed to have a greater effect on proliferation capacity than intrapair weight difference, since proliferation rates varied greatly between pairs but were similar within pairs in both leaner and heavier WD cotwins.

Obesity has been shown to alter the immunophenotype of ASCs [16, 17]; specifically, higher expression of HLA II and CD106 and lower expression of CD29 have been observed in obASCs than in lnASCs [16]. De Girolamo et al. reported lower expression of CD54 and CD34 in obASCs than in lnASCs [17]. On the other hand, it has been reported that obesity of the donor does not affect the CD marker expression of ASCs [26, 44, 45]. In the current study, ASC from all donors showed a characteristic immunophenotype of MSCs.

Interestingly, in our study, the expression of CD146 was lower in ASCs from heavier twins than in ASCs from leaner cotwins. CD146 is considered a marker for pericytes, which are localized around the endothelial layer in capillaries and are involved in angiogenesis and blood vessel homeostasis and functions [46]. Silva et al. [24] reported that stromal vascular fraction (SVF) cells from donors with obesity have higher expression of CD146 and therefore higher levels of pericytes in their SVF. Our results with culture-expanded ASCs are opposing, suggesting that heavier cotwins would have a decreased number of pericytes in their AT. Lower CD146 expression could indicate decreased proangiogenic potential of obASCs. In line with our hypothesis, it was previously reported that obASCs have reduced proangiogenic potential that is mediated by extracellular vesicles derived from obASCs [47]. To study the angiogenic potential of ASCs, we cocultured ASCs with HUVECs and analyzed the formed vascular-like structures. Our results revealed a significantly decreased angiogenic potential of heavier WD twins compared with leaner cotwins. Thus, the acquired weight of a donor has a significant influence on the angiogenic potential of ASCs, which was evidenced in our study as reduced expression of CD146 and impaired capacity to form vascular-like structures in coculture with HUVECs.

The effect of obesity on ASC differentiation capacity has been studied previously, but the results have been contradictory. The obesity of the donor has been shown to reduce the differentiation capacity of isolated ASC [16, 17, 19, 21, 23–25]. In most of the studies, elevated BMI has been linked to compromised osteogenesis [17, 19, 23] and adipogenesis [16, 17, 21, 24, 25]. Opposing results, however, exist where the differentiation capacity has increased [18, 26] or not been affected due to obesity of the donors [19, 27]. In our study, ASCs differentiated toward adipogenic lineages and showed characteristic biological donor variation in differentiation capacity. In addition, a significantly increased number of large lipid droplets formed in heavier cotwins compared with leaner cotwins, which indicates a more prominent adipogenic potential of donors with a higher BMI, as previously reported [18, 26]. Moreover, in a study by Zhu et al., it was shown that ASCs from obese pigs has increased adipogenic differentiation capacity, which was partly linked to upregulation of *TNF* [48]. This result was in line with our study, where ASCs from heavier cotwins had an increased number of large lipid droplets combined with higher expression of proinflammatory *IL-6* and *TNF*. Similarly, Serena et al. reported that obASCs showed higher expression of *TNF* than lnASCs [28]. The increased expression of proinflammatory markers and improved adipogenic differentiation capacity of ASCs seem both to be associated with increased donor weight. It could be speculated that increased *TNF* expression in obASCs may explain the increased adipogenic differentiation capacity of heavier WD cotwins in this study.

The effect of obesity on osteogenic differentiation capacity has shown varying results. A poor capacity of obASCs for osteogenic differentiation has previously been linked with the upregulation of inflammatory transcripts [21]. Furthermore, it has been shown that patients diagnosed with inflammatory diseases have a reduced capacity for osteogenesis and a higher incidence of osteoporosis [49]. In our study, the higher expression of proinflammatory markers did not lead to differences in osteogenic capacity, although three out of four heavier WD twins had reduced mineralization capacity and relative ALP expression compared with leaner WD cotwins. Our study is in line with a previous study showing that donor BMI does not significantly correlate with osteogenic differentiation capacity; however, a tendency for decreased expression of osteogenic markers can be observed [27].

The capacity of ASCs to modulate immune cells and immune responses has made them potential tools for the treatment of chronic inflammatory diseases [9]. In previous studies, obesity has been shown to impair the therapeutic value of ASCs and reduce the immunosuppressive capacity of ASCs by enhancing the proliferation of T cells [28, 29]. These previous studies indicate that ASCs derived from donors with obesity may not be suitable for cell-based therapies, especially for the treatment of autoimmune diseases or inflammatory diseases [29]. Our results contradict these previous observations. All ASCs from the donors, regardless of intrapair weight difference, had low immunogenicity and were capable of immunosuppression. Unexpectedly, heavier cotwins showed a slightly higher but statistically significant capacity for immunosuppression in direct cocultures than leaner cotwins.

In the human body, ASCs faces drastic changes in AT during the development of obesity. These include changes in AT composition, structure and function as well as an increased number of immune cells and proinflammatory cytokines leading to metabolic dysfunction [14]. The inflammatory milieu, hypoxia, and abnormal metabolites in obese AT impair the functions of ASCs, and as a consequence, they gradually become compromised in their regulatory and modulatory functions over time [14]. A relevant question is whether the inflammatory milieu in obese AT will cause permanent changes in ASCs and whether we can observe these changes in vitro after long cell expansion periods. We have previously shown that ASC characteristics, e.g., proliferation, differentiation capacity, and immunophenotype, are modulated by in vitro culture conditions [10]. We speculate that the inflammatory environment of the source AT may affect ASC characteristics in the early phases of in vitro culture, but the effect may be lost in long-term culture.

There were certain limitations in this study. We had a low number of MZ WD twin pairs with small, but significant difference in their BMI. Due to small biopsy size, the expansion of one ASC donor cell line took a longer time compared with other cell lines. That particular ASC donor cell line did not differentiate into adipogenic or osteogenic lineages and detached repeatedly on angiogenesis potential assay reducing the number of donors in differentiation studies, suggesting that the quality of the starting material might have an effect on cell behavior.

In summary, in this study, we observed that the acquired weight of a heavier donor compared to a genetically matched leaner cotwin led to lower expression of the surface marker CD146, impaired angiogenic potential, improved adipogenic differentiation capacity, and higher expression of the proinflammatory marker *TNF.* Our results show that the acquired weight of a donor has a significant influence on ASC characteristics, although all donors were metabolically rather healthy. In addition, heavier donors showed improved suppression capacity compared with leaner donors, while ASC proliferation, osteogenic differentiation capacity, and immunogenicity were similar independent of intrapair weight differences. Our results demonstrate that acquired weight has an influence on certain ASC characteristics independent of predisposing genetics.

## Conclusions

Our results suggest that the acquired weight of a donor may lead to impaired angiogenic potential and increased adipogenic potential of ASCs, independent of genetic background. Considering the clinical use of ASCs, a careful characterization and selection of donors should be carried out, since the weight of a donor may influence the clinical potential of ASCs.

## Supplementary Information

**Additional file 1: Supplementary Tables. Supplementary Table 1.** Medium composition of HS medium (BM), HPL medium, adipogenic medium (AM), osteogenic medium (OM), chondrogenic medium (CM) and endothelial medium (EM). **Supplementary Table 2.** Antibodies of surface protein markers and manufacturers. **Supplementary Table 3.** Forward and reverse sequences, product size and accession numbers of osteogenic and adipogenic gene expression markers. **Supplementary Table 4.** Inflammatory gene expression markers. **Supplementary Table 5.** Surface marker expression of ASCs isolated from MZ twin donors. **Supplementary Table 6.** P-values and statistical tests used. **Supplementary Table 7.** P-values and statistical tests used in proliferation. **Supplementary Table 8.** P-values and statistical tests used in osteogenic and adipogenic differentiation capacity, control condition (BM) vs. differentiation condition (OM/AM). **Supplementary Table 9.** P-values and statistical tests used in immunogenicity, sample vs. control condition (PHA).

**Additional file 2: Figure 1.** Proliferation capacity of ASCs. Pair numbers 1–5. All quantitative data are presented as the mean ± SD.

**Additional file 3: Figure 2.** Osteogenic differentiation capacity of ASCs. (a) Alizarin red (AR) staining, (b) quantified AR staining, (c) alkaline phosphatase (ALP) activity, (d) relative expression of *RUNX2* and (e) *ALP*. Pair number 2–5. All quantitative data are presented as the mean ± SD.

**Additional file 4: Figure 3.** Adipogenic differentiation capacity of ASCs. (a) Oil Red O (ORO) staining, (b) quantified ORO, (c) relative expression of *AP2* and (d) *PPARγ*. Pair numbers 2–5. All quantitative data are presented as the mean ± SD.

**Additional file 5: Figure 4.** (a) Vasculature area and (b) length of vasculature-like structures created by HUVEC in coculture with ASCs. Pair numbers 2–5. All quantitative data are presented as mean ± SD.

**Additional file 6: Figure 5.** (a) Immunogenicity, immunosuppression capacity in (b) direct and (c) indirect cocultures, relative gene expression of (d) *IL-6* and (e) TNF. Pair numbers 1-5. PHA = Phytohemagglutinin-M. All quantitative data are presented as the mean ± SD.

**Additional file 7: Figure 6.** Morphology of ASCs derived from MZ twin pairs. Inset = scale bar 100 µm, larger picture = scale bar 1 mm.

**Additional file 8: Figure 7.** Chondrogenic differentiation capacity of ASCs derived from MZ twin pairs. Alcian blue staining, scale bar 100 µm.

## Data Availability

The datasets generated and/or analyzed during the current study are available from the corresponding author on reasonable request.

## Abbreviations

ASC: Adipose stromal/stem cells
MSC: Mesenchymal stromal/stem cells
ISCT: The International Society for Cellular Therapy
AT: Adipose tissue
obASC: ASC isolated from donors with obesity
lnASC: ASC isolated from lean donors
WD: Weight-discordant
MZ: Monozygotic
BMI: Body mass index
hs-CRP: High-sensitivity C reactive protein
ELISA: Enzyme-linked immunosorbent assay
HDL: High-density lipoprotein
LDL: Low-density lipoprotein
HOMA-IR: Insulin resistance index
HS: Human serum
HPL: Human platelet lysate
PE-Cy7: Phycoerythrincyanine 7
APC: Allophycocyanin
BV711: Brilliant violet 711
FITC: Fluorescein isothiocyanate
CCK8: Cell counting kit-8
DPBS: Dulbecco’s phosphate-buffered solution
OM: Osteogenic medium
AR: Alizarin red
QAR: Quantified alizarin red
ALP: Alkaline phosphatase
AM: Adipogenic medium
ORO: Oil Red O
QORO: Quantified Oil Red O
LD: Lipid droplet
RUNX2: Runt-related transcription factor 2
PPARγ: Peroxisome proliferator activated receptor gamma
AP2: Adipocyte protein 2
RPLP0: Ribosomal protein P0
IL-6: Interleukin 6
TNF: Tumor necrosis factor
GAPDH: Glyceraldehyde-3-phosphate dehydrogenase
HUVEC: Human umbilical vein endothelial cells
EM: Endothelial medium
PBMC: Peripheral blood mononuclear cells
MLR: Mixed lymphocyte reaction
PHA: Phytohemagglutinin-M
SD: Standard deviation
ND: Not detected
SVF: Stromal vascular fraction
