# Supplementary material for: Evaluation of the effect of donor weight on adipose stromal/stem cell characteristics by using weight-discordant monozygotic twin pairs
Source: Stem Cell Res Ther. 2021 Sep 26;12:516. doi: 10.1186/s13287-021-02587-0 (PMC8474937; doi:10.1186/s13287-021-02587-0)
Supplement: Supplementary file 1 — Additional file 1: Supplementary Tables. Supplementary Table 1. Medium composition of HS medium (BM), HPL medium, adipogenic medium (AM), osteogenic medium (OM), chondrogenic medium (CM) and endothelial medium (EM). Supplementary Table 2. Antibodies of surface protein markers and manufacturers. Supplementary Table 3. Forward and reverse sequences, product size and accession numbers of osteogenic and adipogenic gene expression markers. Supplementary Table 4. Inflammatory gene expression markers. Supplementary Table 5. Surface marker expression of ASCs isolated from MZ twin donors. Supplementary Table 6. P-values and statistical tests used. Supplementary Table 7. P-values and statistical tests used in proliferation. Supplementary Table 8. P-values and statistical tests used in osteogenic and adipogenic differentiation capacity, control condition (BM) vs. differentiation condition (OM/AM). Supplementary Table 9. P-values and statistical tests used in immunogenicity, sample vs. control condition (PHA). [file 13287_2021_2587_MOESM1_ESM.docx]

**Supplemental Materials**

**Supplementary Tables**

**Supplementary Table 1.** Medium compositions of HS medium (BM), HPL medium, adipogenic medium (AM), osteogenic medium (OM), chondrogenic medium (CM) and endothelial medium (EM).

| **Medium** | **Reagents** |
| --- | --- |
| **HS medium**  **(BM)** | DMEM/F12 (1:1) (Thermo Fisher Scientific Inc., Carlsbad, CA, https://www.thermofisher.com)  5% Human serum (Paa Laboratories/BioWest)  1% antibiotics (100 U/ml penicillin and 0.1 mg/ml streptomycin; Thermo Fisher Scientific Inc.)  1% L-glutamine (GlutaMAX-100; Thermo Fisher Scientific Inc) |
| **HPL medium** | DMEM/F12 (1:1)  5% Pooled Human Platelet Lysate (Stemulate™; Cook General BioTechnology, Indianapolis, IN, http://www.cookregentec.com)  1% antibiotics  1% L-glutamine |
| **Adipogenic medium**  **(AM)** | DMEM/F12 (1:1)  5% Human serum  1% antibiotics  1% L-glutamine  100 nM Insulin (Gibco)  1 µM Dexamethasone (Sigma)  17 µM Panthothenate (Fluka)  33 µM Biotin (Sigma)  250 µM IBMX for 72 h induction in the beginning of differentiation |
| **Osteogenic medium**  **(OM)** | DMEM/F12 (1:1)  5% Human serum  1% antibiotics  1% L-glutamine  150 µM L-ascorbic acid 2- phosphate (Sigma)  10 mM β-Glyserophosphate (Sigma)  10 nM Dexamethasone (Sigma) |
| **Chondrogenic medium (CM)** | DMEM/F/12 (1:1)  0,3% antibiotics  1% L-glutamine  50 µM L-ascorbic acid 2- phosphate (Sigma)  55 µM sodium pyruvate (Lonza)  1x ITS+ (Sigma)  23 µM L-proline (Sigma)  10 ng/ml TGF-β1 (Sigma) |
| **Endothelial medium**  **(EM)** | EBM-2 (Lonza, Bullet-kit)  2% Human serum (Paa Laboratories/BioWest)  hFGF-B (Lonza, Bullet-kit)  VEGF (Lonza, Bullet-kit)  R3-IGF-1 (Lonza, Bullet-kit)  hEGF (Lonza, Bullet-kit)  GA-1000 (Lonza, Bullet-kit)  HEPARIN (Lonza, Bullet-kit)  Hydrocortisone (Lonza, Bullet-kit)  Ascorbic acid (Lonza, Bullet-kit) |

**Supplementary Table 2.** Antibodies of surface protein markers and manufactures.

| **Marker** | **Surface protein** | **Manufacturer** |
| --- | --- | --- |
| CD14-PE-CF594 | Lipopolysaccharide reseptor | BD Biosciences |
| CD19-PE-Cy7 | B lymphocyte-lineage differentiation antigen | BD Biosciences |
| CD34-PE-CF594 | Hematopoietic progenitor cell antigen 1 | BD Biosciences |
| CD45R0-APC | RO isoform of leucocyte common antigen | BD Biosciences |
| CD54-BV711 | Intercellular adhesion molecule 1 (ICAM-1) | BD Biosciences |
| CD73-PE-Cy7 | Ecto 5’ nucleotidase | BD Biosciences |
| CD90-APC | Thy-1 (T cell surface glycoproteins) | BD Biosciences |
| CD105-FITC | SH-2, endoglin | Immunotools GmbH |
| CD146-PE | Melanoma cell adhesion molecule (MCAM) | BD Biosciences |
| HLA-DR-BV421 | Major histocompatibility class II antigen (MHC-II) | BD Biosciences |
| HLA-ABC-PE | Major histocompatibility class I antigen (MHC-I) | Immunotools GmbH |

**Supplementary Table 3.** Forward and reverse sequences, product size and accession numbers of osteogenic and adipogenic gene expression markers.

| **Name** | **5’-Sequence-3’** | **Product Size (bp)** | **Accession Number** |
| --- | --- | --- | --- |
| *RPLP0* | forward AATCTCCAGGGGCACCATT  reverse CGCTGGCTCCCACTTTGT | 70 | NM_001002 |
| *ALP* | forward CCC CCG TGG CAA CTC TAT CT  reverse GAT GGC AGT GAA GGG CTT CTT | 73 | NM_000478.4 |
| *RUNX2* | forward CTTCATTCGCCTCACAAACAAC  reverse TCCTCCTGGAGAAAGTTTG | 62 | NM_001024630.3 |
| *PPARγ* | forward CAGTGTGAATTACAGCAAACC  reverse ACAGTGTATCAGTGAAGGAAT | 100 | NM_015869 |
| *AP2* | forward GGTGGTGGAATGCGTCATG  reverse CAACGTCCCTTGGCTTATGC | 71 | NM_001442 |

**Supplementary Table 4.** Inflammatory gene expression markers.

| **Detector** | **Gene name** |
| --- | --- |
| **Inflammatory marker** |  |
| IL-6-Hs00985639_m1 | Interleukin 6 |
| TNF-α-Hs-01113624_g1 | Tumor necrosis factor alpha |
| **Housekeeping gene** |  |
| GAPDH Hs02786624_g1 | Glyceraldehyde-3-phosphate dehydrogenase |

**Supplementary Table 5**. Surface marker expression of ASC isolated from MZ twin donors.

| **Twin pair** | **1** | | **2** | | **3** | | **4** | | **5** | |
| --- | --- | --- | --- | --- | --- | --- | --- | --- | --- | --- |
| **CD marker** | leaner | heavier | leaner | heavier | leaner | heavier | leaner | heavier | leaner | heavier |
| **CD14** | 0.3 | 3.1 | 0.5 | 0.2 | 0.4 | 0.3 | 0.3 | 0.2 | 0.3 | 0.2 |
| **CD19** | 1.6 | 3.1 | 1.1 | 1 | 1.5 | 1.6 | 0.9 | 1.1 | 2.9 | 1.8 |
| **CD34** | 2.3 | 9.8 | 5.8 | 7.1 | 2.1 | 7.6 | 2.3 | 1.8 | 8.3 | 5.5 |
| **CD45-RO** | 0.9 | 1.4 | 0.6 | 0.7 | 0.9 | 0.9 | 0.9 | 1.1 | 1 | 0.9 |
| **CD54** | 35.7 | 24.8 | 11.4 | 7.1 | 8.9 | 13.1 | 4 | 4 | 21 | 17.8 |
| **CD73** | 100 | 100 | 100 | 100 | 100 | 100 | 100 | 100 | 99.9 | 99.9 |
| **CD90** | 99.7 | 99.8 | 96.6 | 99.7 | 99.6 | 99.7 | 100 | 100 | 99.7 | 99.7 |
| **CD105** | 99.2 | 99.7 | 99.1 | 95.1 | 99.7 | 99.1 | 99.7 | 99.7 | 99.3 | 99.2 |
| **CD146** | 60.9 | 25.7 | 29.3 | 10.8 | 71.3 | 14.5 | 45 | 33.8 | 5.2 | 5 |
| **HLA-DR** | 1.4 | 0.4 | 0.7 | 0.4 | 6.4 | 0.2 | 0.5 | 0.4 | 0.3 | 0.4 |
| **HLA-ABC** | 99.3 | 99.4 | 98.2 | 99.5 | 99.6 | 99.8 | 99.7 | 99.9 | 99.6 | 99.3 |

**Supplementary Table 6**. P-values and statistical tests used.

| Variable | Weight-discordant pairs (ΔBMI >3 kg/m^2^) | | | | | | | |
| --- | --- | --- | --- | --- | --- | --- | --- | --- |
|  | Leaner co-twin | | vs. | Heavier co-twin | |  |  |  |
|  | mean | SD |  | mean | SD | Test | p- value |  |
| Proliferation 4 d | 0.015 | 0.006 |  | 0.014 | 0.005 | Wilcoxon signed-rank test | 0.585 |  |
| Proliferation 7 d | 0.131 | 0.06 |  | 0.136 | 0.05 | Wilcoxon signed-rank test | 0.893 |  |
| Proliferation 11 d | 0.42 | 0.12 |  | 0.46 | 0.1 | Wilcoxon signed-rank test | 0.5 |  |
| QALP | 5.23 | 4.81 |  | 6.42 | 4.66 | Wilcoxon signed-rank test | 1 |  |
| *ALP* | 3.9 | 1.9 |  | 2.5 | 0.7 | Multilevel mixed-effects regression model test | 0.138 |  |
| *RUNX2* | 4.87 | 2.8 |  | 6.2 | 0.8 | Wilcoxon signed-rank test | 0.465 |  |
| QAR | 1.02 | 0.34 |  | 0.78 | 0.41 | Multilevel mixed-effects regression model test | 0.149 |  |
| QORO (LD area/nuclei (µm^2^) | 39.9 | 29.7 |  | 60.2 | 50.4 | Multilevel mixed-effects regression model test | **0.011** |  |
| *PPARγ* | 6.23 | 2.34 |  | 7.99 | 3.8 | Wilcoxon signed-rank test | 0.465 |  |
| *AP2* | 529 | 488 |  | 1005 | 1002 | Wilcoxon signed-rank test | 0.465 |  |
| Vasculature area (px) | 403061 | 108467 |  | 317381 | 60880 | Multilevel mixed-effects regression model test | **0.039** |  |
| Vasculature length (px) | 35429 | 10679 |  | 26648 | 4817 | Multilevel mixed-effects regression model test | **0.031** |  |
| Immunogenicity | 0.37 | 0.2 |  | 0.32 | 0.15 | Wilcoxon signed-rank test | 0.5 |  |
| Immunosuppression capacity direct | 0.7 | 0.24 |  | 0.58 | 0.16 | Multilevel mixed-effects regression model test | **0.017** |  |
| Immunosuppression capacity indirect | 1.1 | 0.18 |  | 1.02 | 0.2 | Wilcoxon signed-rank test | 0.345 |  |
| *IL-6* | 1.29 | 0.81 |  | 1.52 | 0.83 | Multilevel mixed-effects regression model test | 0.137 |  |
| *TNF* | 1.087 | 0.62 |  | 1.831 | 0.91 | Multilevel mixed-effects regression model test | **0.015** |  |

**Supplementary Table 7**. P-values and statistical tests used in proliferation.

| Variable | Timepoint |  | vs. | Timepoint |  |  |  |
| --- | --- | --- | --- | --- | --- | --- | --- |
|  | mean | SD |  | mean | SD | Test | p- value |
| Proliferation (leaner) | d4 |  |  | d7 |  | Multilevel mixed-effects regression model test |  |
|  | 0.015 | 0.006 |  | 0.131 | 0.06 |  | **<0.001** |
| Proliferation (leaner) | d4 |  |  | d11 |  | Multilevel mixed-effects regression model test |  |
|  | 0.015 | 0.006 |  | 0.42 | 0.12 |  | **<0.001** |
| Proliferation (leaner) | d7 |  |  | d11 |  | Multilevel mixed-effects regression model test |  |
|  | 0.131 | 0.06 |  | 0.42 | 0.12 |  | **<0.001** |
| Proliferation (heavier) | d4 |  |  | d7 |  | Multilevel mixed-effects regression model test |  |
|  | 0.014 | 0.005 |  | 0.136 | 0.05 |  | **<0.001** |
| Proliferation (heavier) | d4 |  |  | d11 |  | Multilevel mixed-effects regression model test |  |
|  | 0.014 | 0.005 |  | 0.46 | 0.11 |  | **<0.001** |
| Proliferation (heavier) | d7 |  |  | d11 |  | Multilevel mixed-effects regression model test |  |
|  | 0.136 | 0.05 |  | 0.46 | 0.11 |  | **<0.001** |

**Supplementary Table 8**. P-values and statistical tests used in osteogenic and adipogenic differentiation capacity, control condition (BM) vs. differentiation condition (OM/AM).

| Variable | Control | | vs. | Differentiation | |  |  |
| --- | --- | --- | --- | --- | --- | --- | --- |
|  | mean | SD |  | mean | SD | Test | p- value |
| Osteogenic capacity (leaner) | BM |  |  | OM |  |  |  |
| QAR | 0.23 | 0.09 |  | 1.02 | 0.34 | Multilevel mixed-effects regression model test | **<0.001** |
| QALP | 0.94 | 0.13 |  | 5.23 | 4.81 | Multilevel mixed-effects regression model test | **<0.001** |
| *ALP* | 1.62 | 2.06 |  | 3.9 | 1.9 | Multilevel mixed-effects regression model test | **<0.001** |
| *RUNX2* | 0.82 | 0.4 |  | 4.87 | 2.8 | Multilevel mixed-effects regression model test | **<0.001** |
| Osteogenic capacity (heavier) | BM |  |  | OM |  |  |  |
| QAR | 0.28 | 0.11 |  | 0.78 | 0.41 | Multilevel mixed-effects regression model test | **<0.001** |
| QALP | 1.0 | 0.17 |  | 6.42 | 4.66 | Multilevel mixed-effects regression model test | **<0.001** |
| *ALP* | 0.42 | 0.24 |  | 2.5 | 0.7 | Multilevel mixed-effects regression model test | **<0.001** |
| *RUNX2* | 0.97 | 0.41 |  | 6.2 | 0.8 | Multilevel mixed-effects regression model test | **<0.001** |
| Adipogenic capacity (leaner) | BM |  |  | AM |  |  |  |
| QORO | 4.74 | 5.42 |  | 39.9 | 29.7 | Multilevel mixed-effects regression model test | **<0.001** |
| *PPARγ* | 1.2 | 0.41 |  | 6.23 | 2.34 | Multilevel mixed-effects regression model test | **<0.001** |
| *AP2* | 0.37 | 0.35 |  | 529 | 488 | Multilevel mixed-effects regression model test | **<0.001** |
| Adipogenic capacity (heavier) | BM |  |  | AM |  |  |  |
| QORO | 5.21 | 5.39 |  | 60.2 | 50.4 | Multilevel mixed-effects regression model test | **<0.001** |
| *PPARγ* | 1.43 | 0.63 |  | 7.99 | 3.8 | Multilevel mixed-effects regression model test | **<0.001** |
| *AP2* | 0.23 | 0.16 |  | 1005 | 1002 | Multilevel mixed-effects regression model test | **<0.001** |

**Supplementary Table 9**. P-values and statistical tests used in immunogenicity, sample vs. control condition (PHA).

| Variable | Sample |  | vs. | Control |  |  |  |
| --- | --- | --- | --- | --- | --- | --- | --- |
|  | mean | SD |  | mean | SD | Test | p- value |
| Immunogenicity | Leaner |  |  | PHA |  | Multilevel mixed-effects regression model test |  |
|  | 0.37 | 0.2 |  | 7.12 | 1.22 |  | **<0.001** |
| Immunogenicity | Heavier |  |  | PHA |  | Multilevel mixed-effects regression model test |  |
|  | 0.32 | 0.15 |  | 7.12 | 1.22 |  | **<0.001** |
