# Supplementary figures and images for: Evaluation of the effect of donor weight on adipose stromal/stem cell characteristics by using weight-discordant monozygotic twin pairs
Source: Stem Cell Res Ther. 2021 Sep 26;12:516. doi: 10.1186/s13287-021-02587-0 (PMC8474937; doi:10.1186/s13287-021-02587-0)

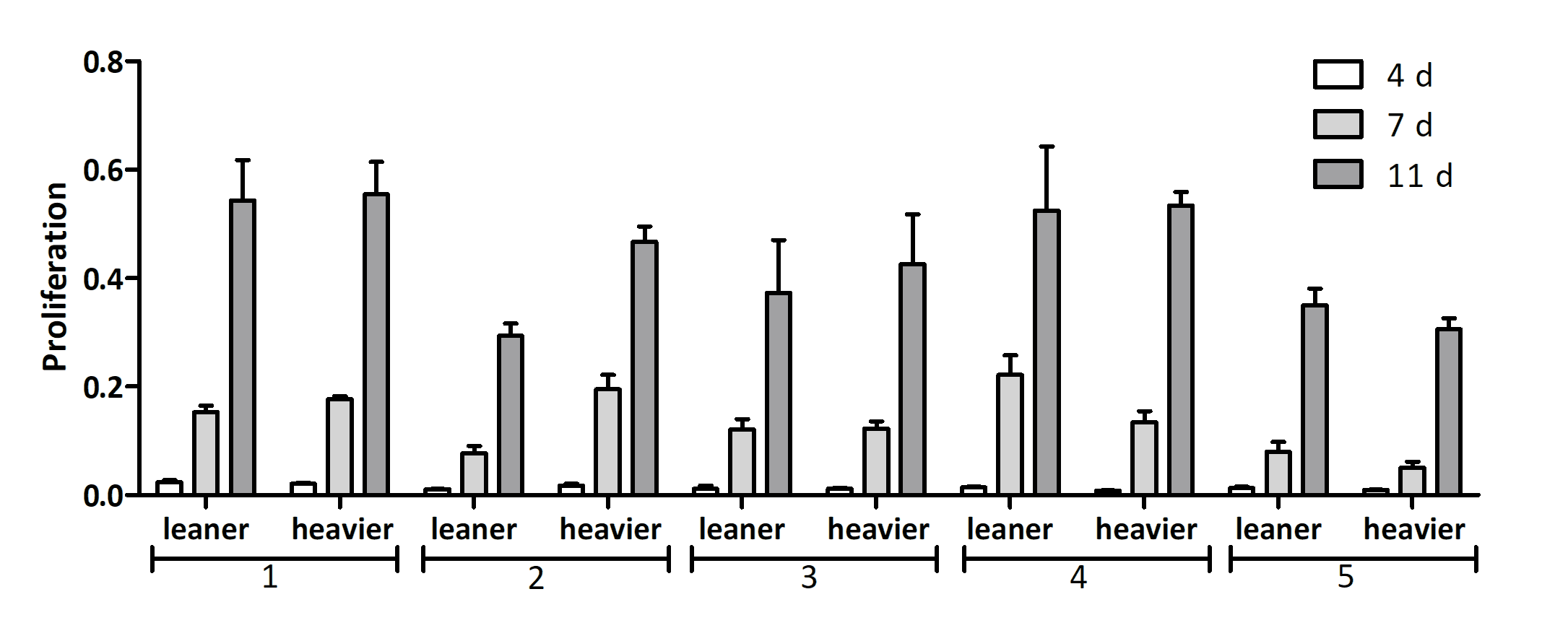

Supplement: Supplementary file 2 — Additional file 2: Figure 1. Proliferation capacity of ASCs. Pair numbers 1–5. All quantitative data are presented as the mean ± SD. [file 13287_2021_2587_MOESM2_ESM.tif]

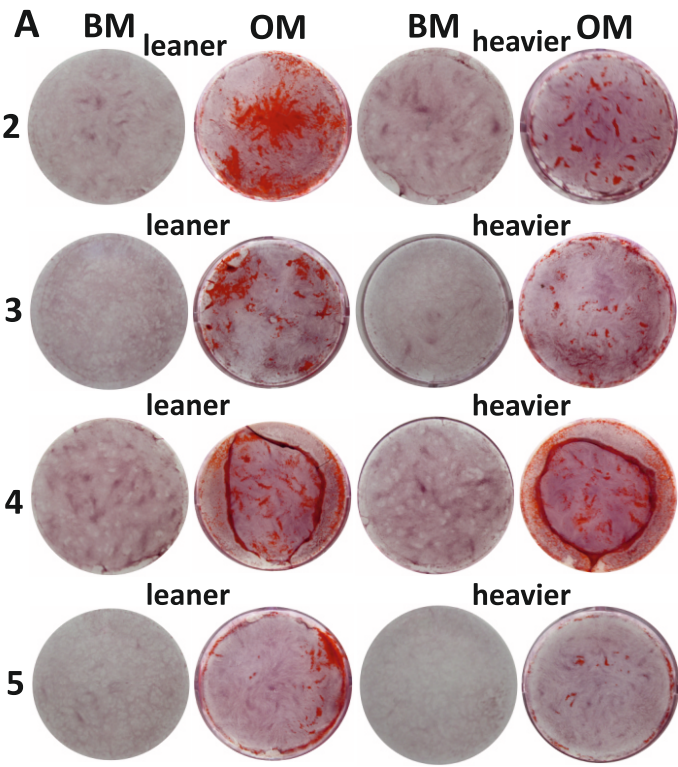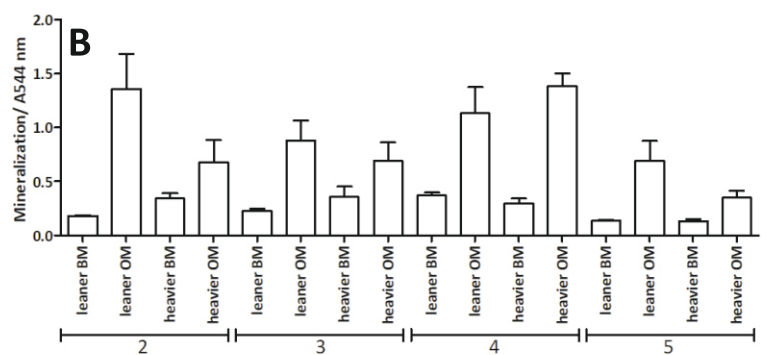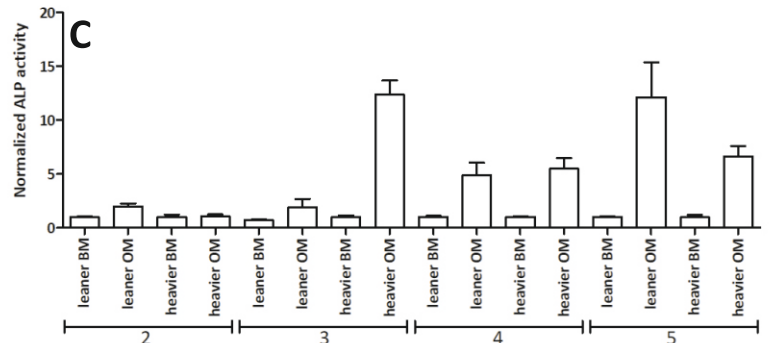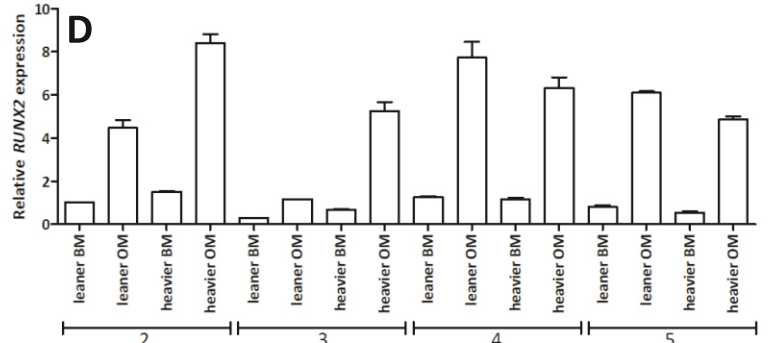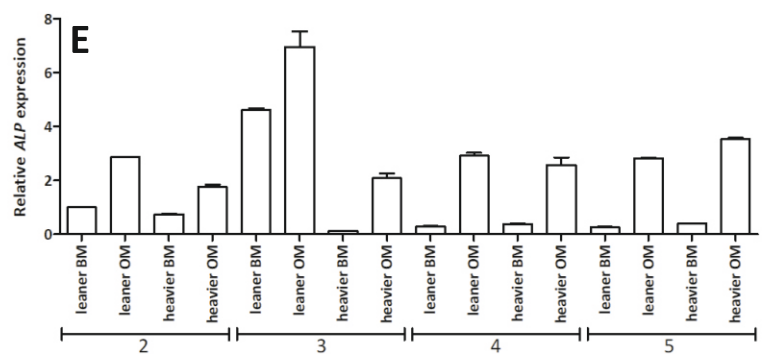

Supplement: Supplementary file 3 — Additional file 3: Figure 2. Osteogenic differentiation capacity of ASCs. (a) Alizarin red (AR) staining, (b) quantified AR staining, (c) alkaline phosphatase (ALP) activity, (d) relative expression of RUNX2 and (e) ALP. Pair number 2–5. All quantitative data are presented as the mean ± SD. [file 13287_2021_2587_MOESM3_ESM.pdf]

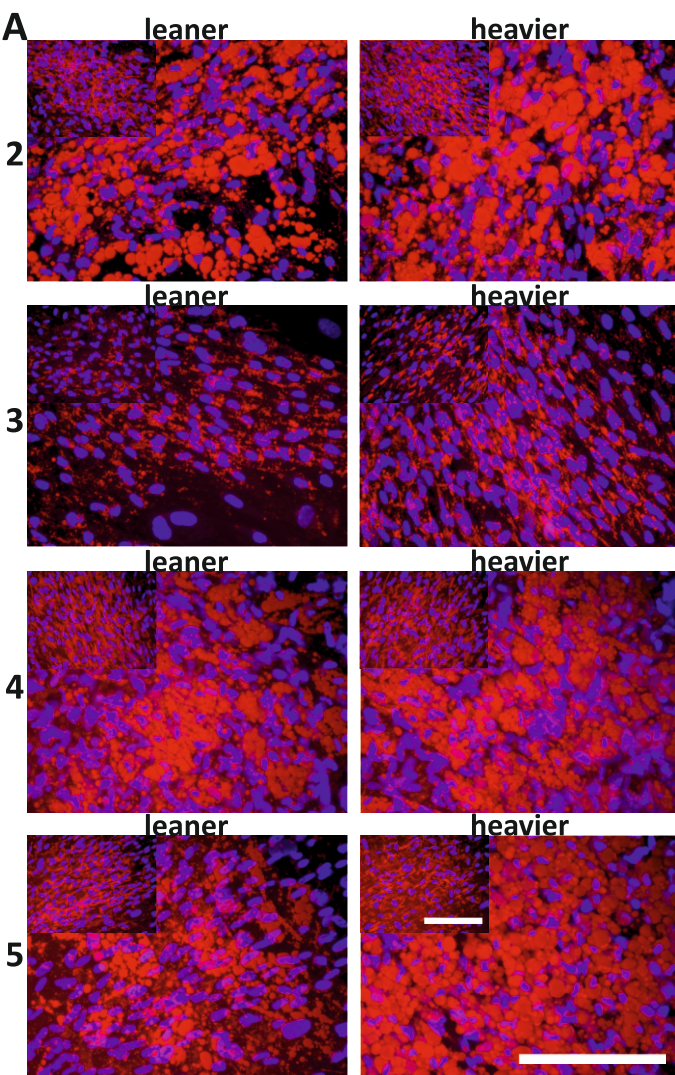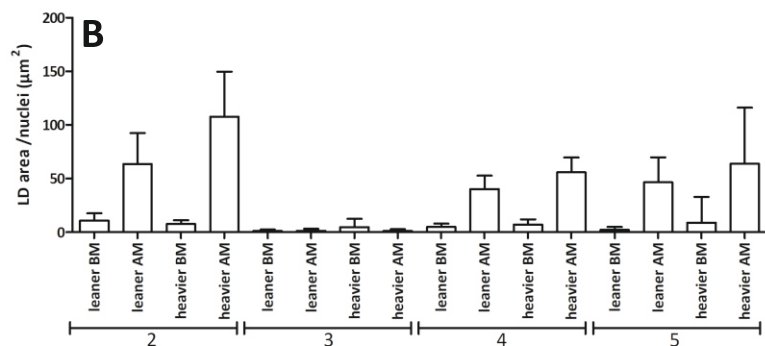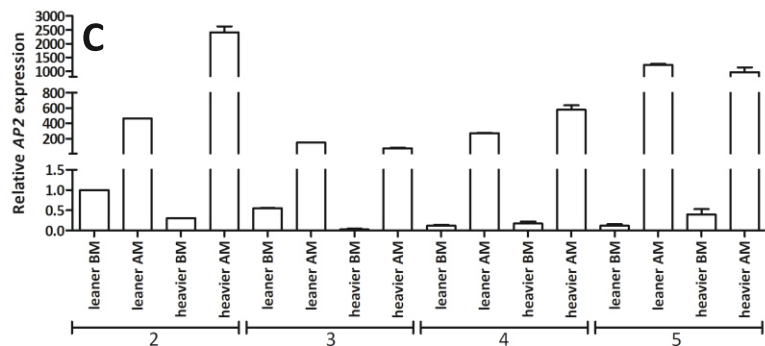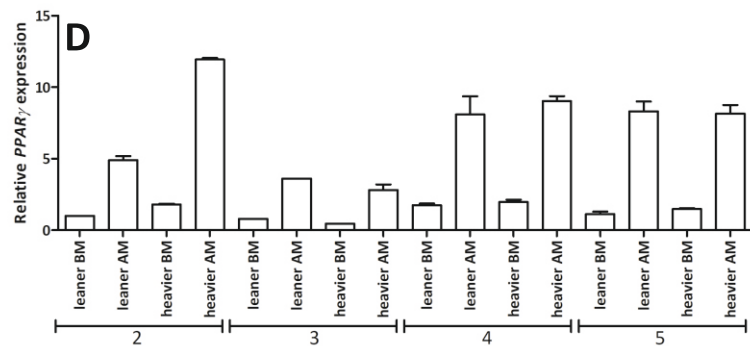

Supplement: Supplementary file 4 — Additional file 4: Figure 3. Adipogenic differentiation capacity of ASCs. (a) Oil Red O (ORO) staining, (b) quantified ORO, (c) relative expression of AP2 and (d) PPARγ. Pair numbers 2–5. All quantitative data are presented as the mean ± SD. [file 13287_2021_2587_MOESM4_ESM.pdf]

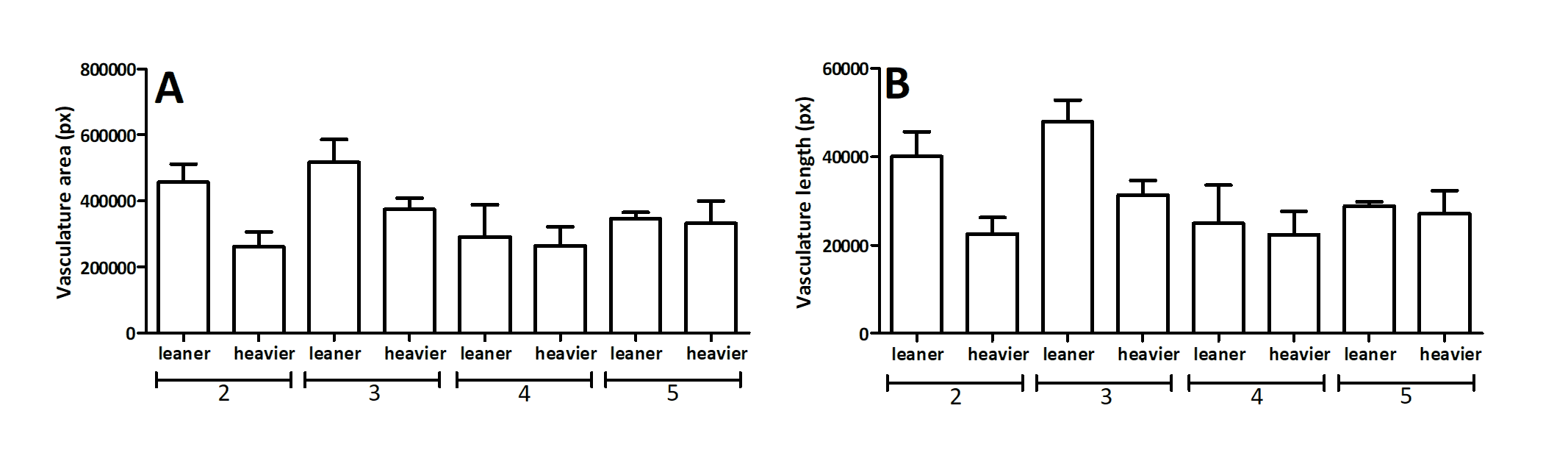

Supplement: Supplementary file 5 — Additional file 5: Figure 4. (a) Vasculature area and (b) length of vasculature-like structures created by HUVEC in coculture with ASCs. Pair numbers 2–5. All quantitative data are presented as mean ± SD. [file 13287_2021_2587_MOESM5_ESM.tif]

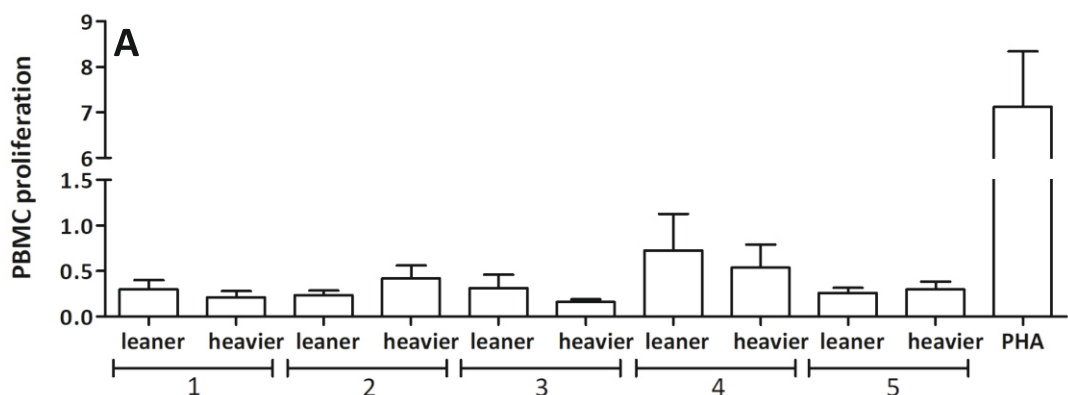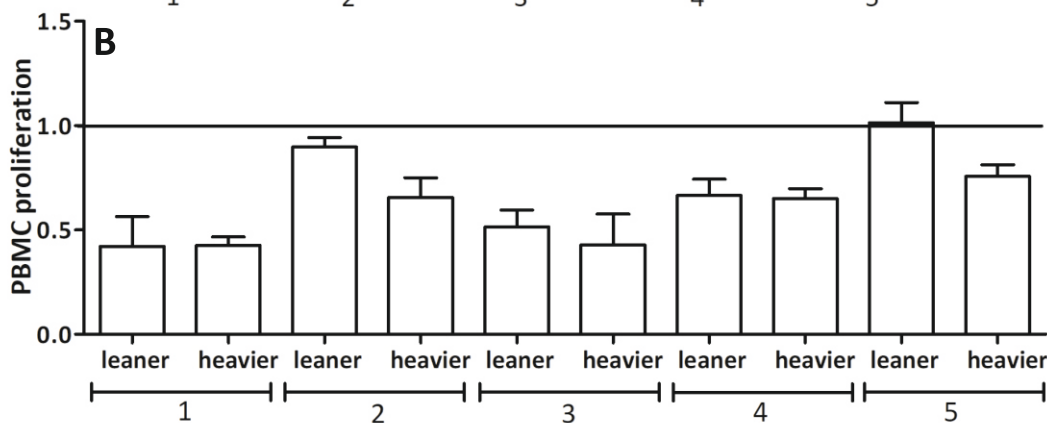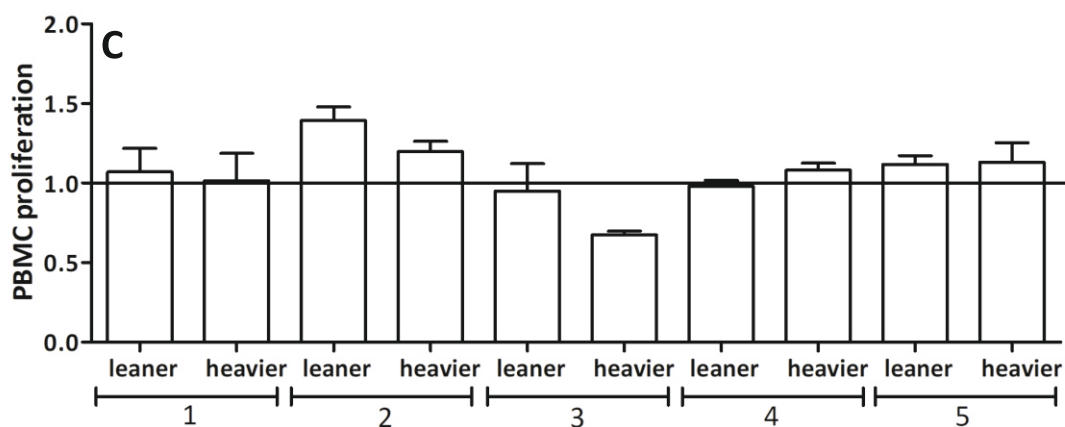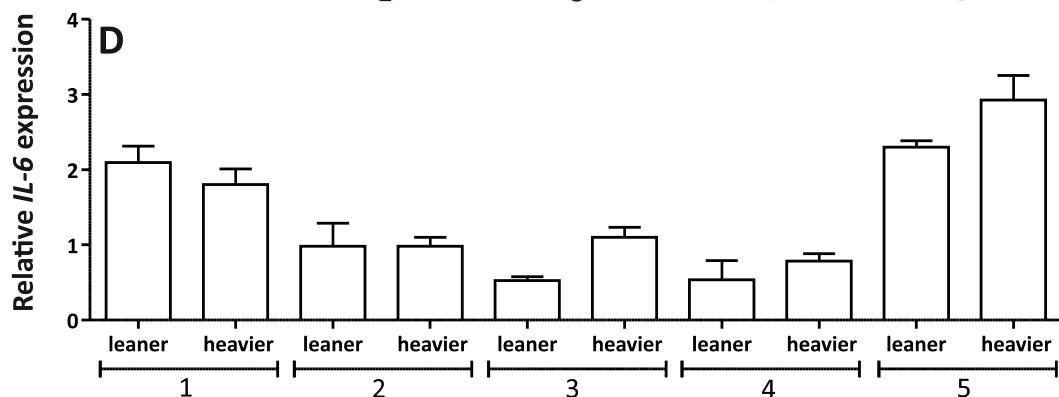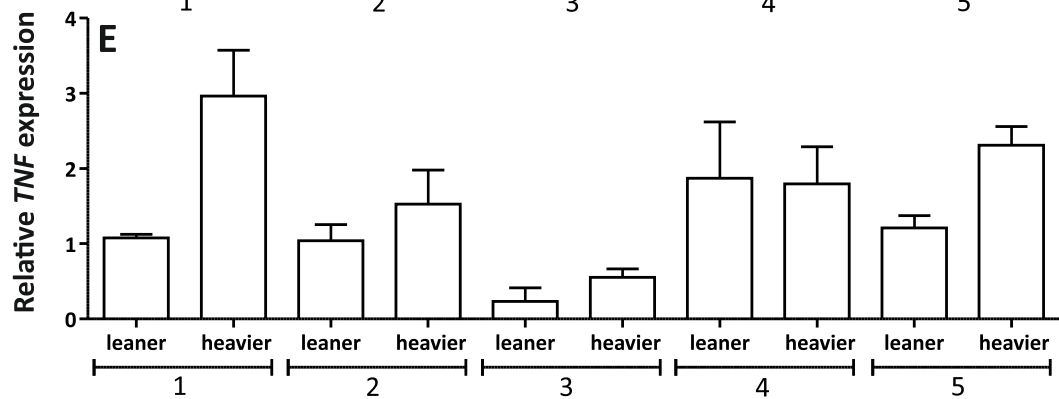

Supplement: Supplementary file 6 — Additional file 6: Figure 5. (a) Immunogenicity, immunosuppression capacity in (b) direct and (c) indirect cocultures, relative gene expression of (d) IL-6 and (e) TNF. Pair numbers 1-5. PHA = Phytohemagglutinin-M. All quantitative data are presented as the mean ± SD. [file 13287_2021_2587_MOESM6_ESM.pdf]

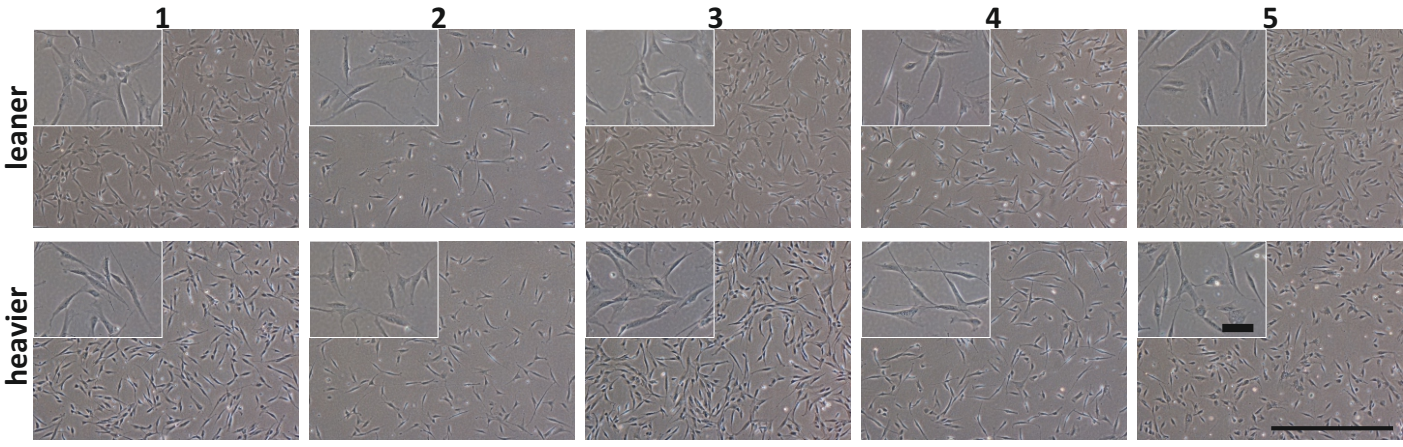

Supplement: Supplementary file 7 — Additional file 7: Figure 6. Morphology of ASCs derived from MZ twin pairs. Inset = scale bar 100 µm, larger picture = scale bar 1 mm. [file 13287_2021_2587_MOESM7_ESM.pdf]

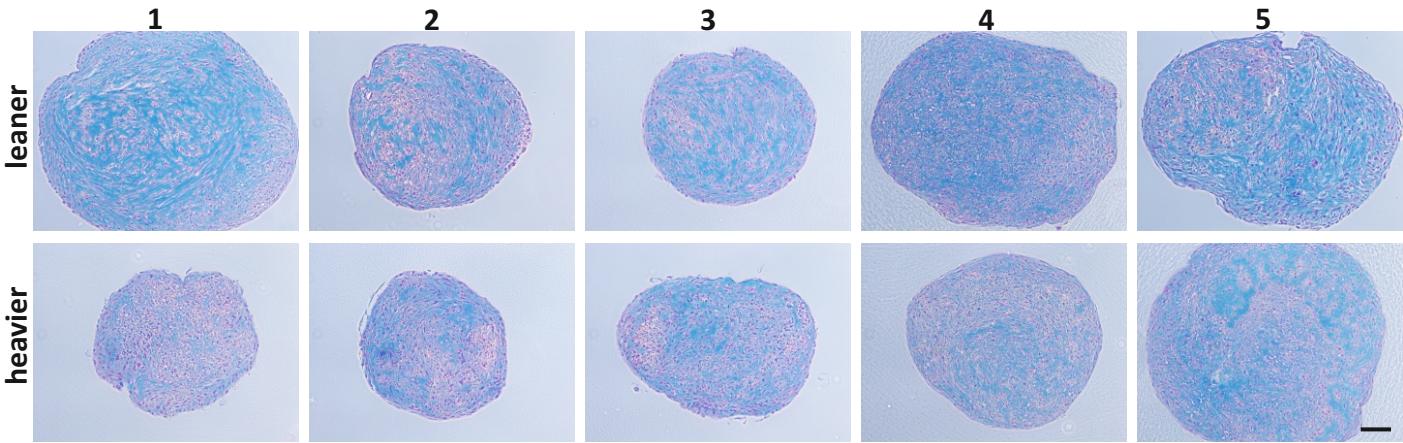

Supplement: Supplementary file 8 — Additional file 8: Figure 7. Chondrogenic differentiation capacity of ASCs derived from MZ twin pairs. Alcian blue staining, scale bar 100 µm. [file 13287_2021_2587_MOESM8_ESM.pdf]
